# The Origin of Syphilis

**Published:** 1905-02

**Authors:** 


### THE ORIGIN OF SYPHILIS.

METCHNIKOF and Roux have for some time past been conducting experiments on the communicability of syphilis to anthropoid apes. Inoculations have been made on chimpanzees of the watery discharge from hard chancres with the production of specific lesions. The material inoculated was filtered through a filter of ascertained transmissibility to various bacterial forms. The filtrate lost its capacity for inoculation on being heated and did not do so when suspended in glycerine, and immediately transferred. These observations are not without significance as they show the relative size of the infecting body from the character of the filter used; the fact that virulence was destroyed by heat and not affected by the germicidal power of glycerine. Immunizing investigations from alternation of the filtrate and from material obtained from inoculation of simians lower than the anthropoid were without result. This is the direction that the further pursuit of the subject is destined to take. Some member of the monkey genus far enough removed in blood relationship to man to act as the immunizing agent is the especial object of search.

The discovery of a specific micro-organism of syphilis would not be so great an advance as the establishment of immunity by an antitoxin; for the specific character of the disease has been theoretically assumed and practically regarded for a long time. The isolation of a known form would merely verify a thoroughly established assumption. A parallel may be made in the instance of the gonococcus which was forecasted until Neisser actually demonstrated it and the resultant good consisted in that to be derived from definite rather than presumptive etiologic conception. The analogy ends here naturally as one may look in vain for immunity from a locally acting disease.

